# Multiple haplotypes of *Chelonia mydas* juveniles in a threatened *hotspot* area in Southern Brazil

**DOI:** 10.1590/1678-4685-GMB-2020-0410

**Published:** 2021-08-30

**Authors:** Camila Satie Savada, Laura Prosdocimi, Camila Domit, Fernanda Simões de Almeida

**Affiliations:** 1 Universidade Estadual de Londrina, Departamento de Biologia Geral, Laboratório de Genética e Ecologia Animal, 86057-970, Londrina, PR, Brazil.; 2 Ministry of Agriculture, Livestock and Fisheries, Buenos Aires, Argentina.; 3 Universidade Federal do Paraná, Laboratório de Ecologia e Conservação, Centro de Estudos do Mar, Pontal do Paraná, 83255-000, Paraná, PR, Brazil.

**Keywords:** Threatened species, foraging area, genetic diversity, mitochondrial DNA, mixed stocks

## Abstract

Mixed stocks are described for *Chelonia mydas* and the frequency of haplotypes in feeding areas can aid understanding of the genetic and ecological diversity, since with this information it is possible to identify the origin of the individuals. The current study aims to characterize and compare genetic diversity along the coast of Paraná with 17 other feeding areas in the Atlantic Ocean. A total of 285 samples from juveniles were DNA sequenced in the control region, resulting in the identification of 12 haplotypes, with a predominance of the CMA8 haplotype (69%) and the first registration of CMA23. For the study subjects, haplotypic and nucleotide diversity were 0.469 ± 0.032 and 0.00189 ± 0.00020, respectively, and comparisons with other feeding areas presented significant values for the majority of FST and ΦST. The results point to the importance of this region and provide evidence that over the years a mixed stock has used the region as a feeding area. This variation could be related to sea currents, climatic changes, and oceanographic characteristics that may alter the availability of food, water temperature, and the presence of turtles. The current results can be considered in conservation plans for *Chelonia mydas*.

## Introduction

The green turtle, *Chelonia mydas,* is affected by multiple threats, and thousands of juveniles are found stranded dead along the Brazilian coast annually (Monteiro et al., 2016; Tagliolatto et al., 2019; Cantor et al., 2020). However, although the species has circumglobal distribution and has been classified as ‘endangered’ by the IUCN (Seminoff, 2004), for the Southwestern Atlantic Ocean, including Brazilian waters, the management unit was recently updated to ‘least concern’ (IUCN, 2019). The IUCN assessment is based primarily on criteria focusing on genetic population stocks and adult abundance estimates at rookery sites, and juvenile mortality is not considered for classifying the risk assessment. Nevertheless, identifying the geographic origin of juveniles is crucial for understanding connectivity and for planning conservation strategies (Wildermann et al., 2018). 

The green turtle exhibits migration and habitat use patterns that are associated with different ocean zones, depending on the development phase (stage of the animal’s life cycle) and genetic origin. The species uses pelagic and oceanic areas during, approximately, its first 2 or 3 years of life, and then moves to demersal and neritic areas in its juvenile and adult phases (Musick and Limpus, 1997). Individuals from different nesting areas congregate in feeding areas, and mixed genetic stocks are recorded in these specific areas. The stocks are identified because they feature specific mitochondrial DNA (mtDNA) haplotypes for each nesting site (Naro-Maciel et al., 2007; 2008; Shamblin et al., 2012); a pattern which is a consequence of the philopatric behavior described for males and females (Jensen *et al*., 2013).

Between 2-4 years of age, juvenile green turtles are recruited from oceanic to coastal zones, and different factors (e.g. ocean currents, genetic origin) influence migratory patterns (Naro-Maciel *et al*., 2014; Andrade et al., 2016; Coelho et al., 2018). Ontogenetic alterations, including morphological and ecological patterns (Gama et al., 2016; Coelho *et al*., 2018), are also associated with diversity in the genetic stocks (Naro-Maciel *et al*., 2016). Studies on the origin of these haplotypes, their presence and frequency in different feeding areas, and the potential new migration routes followed by individuals provide crucial information, both ecological and related to the biogeography of the species. These studies help in establishing and evaluating management and conservation plans aimed at maintaining the genetic and ecological diversity of species (Bowen and Karl, 2007).

Juveniles from different nesting sites are recorded using the Southern and Southeastern coasts of Brazil (Proietti et al., 2012; Jordão et al., 2015) for feeding and development (Almeida et al., 2011; Wallace et al., 2011; Andrade et al., 2016; Gama et al., 2016; Lenz et al., 2017). Off the Paraná coast, in Southern Brazil, juvenile green turtles occur annually in waters of the continental shelf, estuaries, and coastal islands (Guebert-Bartholo et al., 2011; Gama *et al*., 2016). This region is an important feeding area for juveniles, aggregating individuals aged 2-8 years (Andrade *et al*., 2016), migrating from multiple areas (Carman et al., 2012; Fuentes et al., 2020); however, the area presents high mortality rates and is an anthropogenically threatened *hotspot* (Cantor et al., 2020; Fuentes *et al*., 2020).

Anthropogenic activities interfere in the life cycle stages of sea turtles by causing loss of spawning and feeding areas, increasing interaction with fisheries and mortality, and also contributing to habitat degradation, through discarded pollutants and non-biodegradable waste. Therefore, the Brazilian national plan for sea turtle conservation highlights the importance of increasing scientific and management efforts in regions such as Southeastern and Southern Brazil, where the coastal zone is intensely used for activities that are potentially hazardous to sea turtles and their habitats (Sanches, 1999; Fuentes et al., 2020). Improvement in ecological and genetic knowledge will support conservation plans and measures for establishing active initiatives that reduce the threat of exposure and impacts on the species (Mazaris et al., 2009; López-Barrera et al., 2012). Particularly for the immature life stages of sea turtles, studies on population dynamics and survivorship can contribute information on threats and add support to conservation plans (Wilderman *et al*., 2018). 

Evaluating the genetic diversity in feeding areas in Southern Brazil and the variations in different genetic contributions over time is fundamental to understanding and mapping which genetic stocks are most impacted. This knowledge is crucial to assess genetic variability, which includes the potential resilience of a species to continuous degradation of marine ecosystems. Therefore, the present study aimed primarily to characterize green turtles stranded on the Paraná coast from 2007 to 2014 by studying their mtDNA. The secondary objective of this study was to compare the diversity in this area with that recorded in other feeding areas in the Atlantic Ocean.

## Material and Methods

### Area of the study

The Paraná coast in Southern Brazil (north 25°13’ S, 48°1’ W; south 25°58’ S, 48°35’ W), comprises a remnant of Atlantic Forest and a UNESCO Natural World Heritage site (Lana et al., 2000). It is the transition zone between the tropical and temperate areas of the Southwestern Atlantic Ocean and is under the influence of the warm-water Brazil Current and cold-water Malvinas Current (Miranda et al., 2015). This area is composed of estuarine areas, bays, and a shallow continental shelf which are influenced by the El Niño and La Niña weather phenomena and strongly influenced by rainfall (Vanhoni and Mendonça, 2008).

Estuarine areas are crucial because of their ecological role and the Paraná Estuarine Complex is an important foraging and migratory area for juvenile sea turtles. However, the region presents intensive human activity (marine traffic, ports, artisanal fisheries, urban areas, and industrial zones), exposing the species to multiple threats (Fuentes et al., 2020).

### Sampling site

**Figure 1 ‒:**
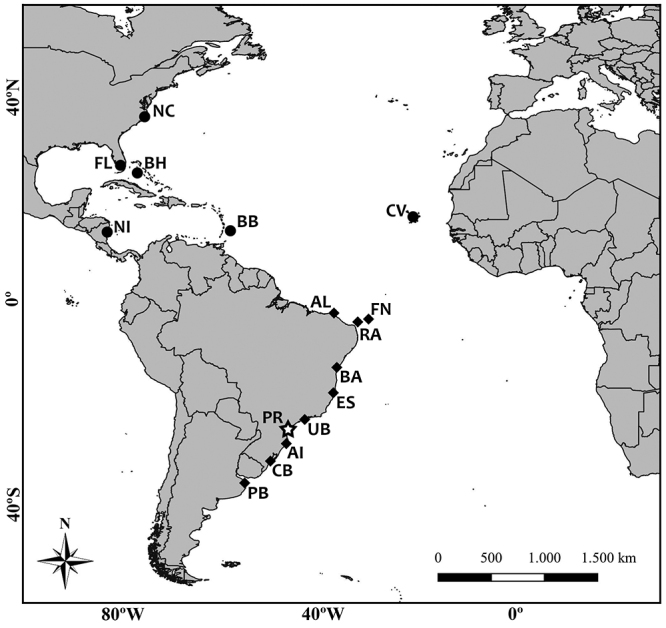
Map of the sample location on the coast of Paraná (PR), Southern Brazil (symbolized by a star). The other feeding areas of the Atlantic Ocean, the North Atlantic group, including North Carolina/USA (NC), Florida/USA (FL), Bahamas (BH), Nicaragua (NI), Barbados (BB), and Cape Verde (CV) indicated by circles; and the South Atlantic group, including Almofala/BR (AL), Fernando de Noronha/BR (FN), Rocas Atoll/BR (RA), Bahia/BR (BA), Espírito Santo/BR (ES), Ubatuba/BR (UB), Arvoredo Island/BR (AI), Cassino Beach/BR (CB), and Buenos Aires Province/AR (PB) represented by lozenges.

The individuals were found either stranded dead, debilitated, or accidentally captured in fishing nets. Samples of liver, epithelial or muscular tissues from juvenile green turtles were collected along the Paraná coast throughout the year from 2007 to 2014 (Figure 1).

The collected tissues were identified according to the animal’s stage of decomposition, adapted from Geraci and Lounsbury (2005), and stored in 100% ethanol until sample preparation for molecular analysis. The curved carapace length (CCL, cm) was measured in all individuals with complete bodies (Wyneken, 2001).

### Molecular techniques

Genomic DNA was extracted according to the phenol-chloroform protocol described by Almeida et al. (2001). The amplification of the control mtDNA region was performed using primers LCM15382 and H950 (Abreu-Grobois et al., 2006), with a final volume of 15 μL (1× PCR buffer, 0.2 mM of each dNTP, 1.5 mM MgCl_2_, 0.5 μM of each primer, 3 U of Taq DNA polymerase [Invitrogen] and 5-50 ng of the DNA sample). The mixture was incubated at 94°C for 5 min; followed by 35 cycles of 40 s at 94°C, 30 s at 51°C, and 1 min at 72°C; and finally, a further 9 min at 72°C. The samples amplified successfully were read in an ABI PRISM 3500xL automatic sequencer (Applied Biosystems).

### Data analysis

The quality of the sequences was checked using the Electropherogram Quality Analysis online software (Togawa and Brigido, 2003). The alignment and manual edition of sequences were performed on MEGA6 (Tamura et al., 2013). The haplotypes in this study were identified according to the Archie Carr Center for Sea Turtle Research database (available online from http://accstr.ufl.edu/genetics.html). Haplotype diversity (*h*), nucleotide diversity (π), and haplotype counts were calculated using DnaSP 5.1 software (Librado and Rozas, 2009).

For comparisons of genetic diversity with other feeding areas of the Southwestern Atlantic Ocean, previously published results were considered, and the 17 included feeding areas were divided into two groups: i) the North Atlantic group, comprising the areas of North Carolina/USA (NC, Bass et al., 2006), Florida/USA (FL, Bass and Witzell, 2000), Bahamas (BH, Lahanas et al., 1998), Nicaragua (NI, Bass *et al*., 1998), Barbados (BB, Luke et al., 2004), and Cape Verde (CV, Monzón-Argüello et al., 2010) and ii) the South Atlantic group, comprising the areas of Almofala/BR (AL, Naro-Maciel et al., 2007), Fernando de Noronha/BR (FN, Naro-Maciel *et al*., 2012), Rocas Atoll/BR (RA, Naro-Maciel *et al*., 2012), Bahia/BR (BA, Naro-Maciel *et al*., 2012), Espírito Santo/BR (ES, Naro-Maciel *et al*., 2012), Ubatuba/BR (UB, Naro-Maciel *et al*., 2007), Paraná/BR (PR, this study), Arvoredo Island/BR (AI, Proietti et al., 2012), Cassino Beach/BR (CB, Proietti *et al*., 2012), and the Province of Buenos Aires/AR (PB, Prosdoscimi *et al*., 2012).

The level of total genetic divergence among sub-populations, according to haplotype frequency (F_ST_) and the Kimura 2P genetic distance parameters (Kimura, 1980) (Φ_ST_), was calculated using Arlequin 3.5.2.2 software (Excoffier and Lischer, 2010). The same software was used to execute the AMOVA to estimate genetic variance partitioning between the stipulated populations and groups, with significance estimates of 5,000 permutations and *p* ≤ 0.05. The AMOVA was executed considering only individuals from the Paraná coast, separated by year of sample collection, using the same criteria for the estimates.

## Results

In the sampling period, approximately 1500 sea turtles were recorded along 40 km of the Paraná coast and estuarine areas according to researchers at Laboratório de Ecologia e Conservação (CEM-UFPR). Tissue samples for genetic analysis were collected from 436 green turtles and DNA samples were sequenced in 285 individuals (~65%). From the mtDNA samples analyzed, 12 haplotypes were defined according to a 485pb region, and 11 of these haplotypes presented 14 polymorphic sites between them, whereas one haplotype (CMA42) had a 4pb insertion/deletion (indel).

Haplotype CMA8 was the most frequently observed haplotype, identified in 195 individuals (69%), followed by CMA5 in 52 individuals (18%). The other 10 haplotypes occurred with a frequency of less than 5%, with only haplotype CMA8 being identified in all years (Table 1).

**Table 1 ‒:** Number of juveniles green turtle from each haplotype in each sampling year (N), with the CCLs (cm) or mean CCLs (cm) (±SD); number of haplotypes per year (Nº hap); haplotype diversity (*h*) and nucleotide diversity (π).

	2007	2008	2009	2010	2011	2012	2013	2014	Total
CMA3			1 (49.5)			1 (35.2)			2
CMA5		7 (35.7 ± 8.1)	8 (36.9 ± 2.7)	7 (34.5 ± 1.5)	5 (44.9 ± 12.2)	18 (35.2 ± 3.1)	2 (31.1 ± 5.0)	5 (36.1 ± 5.8)	52
CMA6				1 (37.5)	2 (37.5 ± 4.9)	2 (37.1 ± 3.4)	2 (36.8 ± 1.1)		7
CMA8	3 (42.5 ± 11.1)	8 (40.3 ± 7.6)	24 (38.5 ± 6.0)	11 (37.4 ± 4.4)	32 (41.3 ± 8.4)	46 (42.4 ± 10.7)	25 (40.0 ± 4.4)	46 (41.3 ± 4.8)	195
CMA9		1 (40.5)				1 (39.3)	2 (46 ± 2.4)	2 (43.4 ± 0.2)	6
CMA10	1 (47.0)							4 (41.9 ± 3.9)	5
CMA23		1 (45.5)							1
CMA24			1 (37.5)	1 (38.0)			1 (55.3)	1 (55.5)	4
CMA32		1 (37.0)							1
CMA39					1 (45.0)	2 (41.3 ± 2.5)			3
CMA42						4 (42.4 ± 6.9)	1 (31.8)	1 (40.2)	6
CMA46					1 (38.5)		1 (32.3)	1 (43.0)	3
N	4	18	34	20	41	74	34	60	285
Nº hap	2	5	4	4	5	7	7	7	
*h*	0.500 (0.265)	0.680 (0.074)	0.458 (0.083)	0.600 (0.077)	0.382 (0.091)	0.489 (0.055)	0.415 (0.104)	0.380 (0.077)	0.469 (0.032)
*π*	0.00104 (0.00055)	0.00279 (0.00036)	0.00264 (0.00094)	0.00225 (0.00029)	0.00132 (0.00033)	0.00227 (0.00047)	0.00116 (0.00034)	0.00118 (0.00028)	0.00189 (0.00020)

The CCLs of the analyzed individuals ranged from 27.5 to 47.6 cm (mean±SD = 39.95 ± 7.32 cm), indicating that all individuals are classified as juveniles (<65 cm CCL) (Bresette *et al*., 2010). No variation was observed among the mean CCLs over the years (*p* = 0.809), and no differences were detected among the mean CCLs of individuals identified as having different haplotypes (*p* = 0.114).

However, it was observed that the diversity of haplotypes is affected by the number of samples collected in the same year; 2012 (n = 74) had the greatest number of samples, with seven (7) identified haplotypes, whereas 2007 (n = 4) had the smallest number of samples, with only two (2) identified haplotypes (Table 1). On the other hand, even though the year 2008 had few samples, haplotypes CMA23 and CMA32 were sampled only in that year. The AMOVA revealed that the difference between sampling years was 3.44% and within years was 96.56%. Haplotype (*h*) and nucleotide (π) diversities were 0.469 ± 0.032 and 0.00189 ± 0.00020, respectively (Table 2).

**Table 2:** ‒ Number of green turtles per haplotype, haplotype diversity (*h*) and nucleotide diversity (*π*) of the mtDNA control region of green sea turtles present in different feeding areas in the Atlantic Ocean. NC = North Carolina/USA; FL = Florida/USA; BH = Bahamas; NI = Nicaragua; BB = Barbados; CV = Cape Verde; AL = Almofala; FN = Fernando de Noronha/BR; RA = Rocas Atoll/BR; BA = Bahia/BR; ES = Espírito Santo/BR; UB = Ubatuba/BR; PR = Paraná/BR; AI = Arvoredo Island/BR; CB = Cassino Beach/BR; PB = Province of Buenos Aires/AR; N = Number of samples; Nº hap = Number of haplotypes; *h* = Haplotype diversity; *π* = Nucleotide diversity. Standard deviations are presented in parentheses.

	North Atlantic							South Atlantic										
	NC	FL	BH	NI	BB	CV	Total	AL	FN	RA	BA	ES	UB	PR	AI	CB	PB	Total
CMA1	34	12	2		7	1	56		2	1								3
CMA2	2	1					3											
CMA3	43	43	62	54	21	2	225	18	4	8		2	2	2	1			37
CMA5	5	3	10	6	13	23	60	28	52	21	14	47	14	52	25	20	20	293
CMA6						1	1	3	4	2	2	6		7	2	2	2	30
CMA8	7		1		14	17	39	53	46	36	23	87	83	195	70	62	59	714
CMA9					1		1	3		7	3	6	4	6	5	3	5	42
CMA10					2		2	4	4	3		3	3	5	2	1	1	26
CMA15	1						1											
CMA16	2						2	1										1
CMA17					1		1		1									1
CMA18	3	2					5											
CMA20			1				1											
CMA21			3				3	1										1
CMA22	2	1			1		4											
CMA23									1		2	2		1	3	2		11
CMA24								1	1		1	2	2	4	3	2	1	17
CMA25																1		1
CMA26	2						2											
CMA27	2						2											
CMA28	3						3											
CMA32								1	1			2	2	1	1	3	2	13
CMA36																2		2
CMA39														3	1		1	5
CMA42								2	1					6	1	1	2	13
CMA44								1					1					2
CMA45								1							1	2		4
CMA46													1	3				4
CMA55													1					1
N	106	62	79	60	60	44		117	117	78	45	157	113	285	115	101	93	
Nº hap	12	6	6	2	8	5		13	11	7	6	9	10	12	12	12	9	
*h*	0.729	0.485	0.370	0.183	0.773	0.588		0.701	0.642	0.703	0.647	0.603	0.446	0.469	0.572	0.561	0.525	
	(0.030)	(0.067)	(0.065)	(0.062)	(0.028)	(0.045)		(0.032)	(0.027)	(0.037)	(0.053)	(0.00095)	(0.056)	(0.032)	(0.045)	(0.051)	(0.052)	
*π*	0.00530	0.00331	0.00642	0.00381	0.01027	0.00419		0.00655	0.00415	0.00551	0.00242	0.00262	0.00205	0.00189	0.00234	0.00199	0.00193	
	(0.00078)	(0.00103)	(0.00113)	(0.00129)	(0.00036)	(0.00107)		(0.00073)	(0.027)	(0.00086)	(0.00023)	(0.00030)	(0.00044)	(0.00020)	(0.00033)	(0.00021)	(0.00021)	

Concerning haplotype occurrence in the 17 Southwestern Atlantic Ocean feeding areas and their classification into two major groups (North and South Atlantic), the AMOVA results indicated that the greatest difference is actually between the groups, whereas the smallest difference is between feeding areas within the groups (Table 3). Comparing all the feeding areas, 81 of 120 F_ST_ comparisons and 73 of 120 Φ_ST_ comparisons were statistically significant, considering *p* ≤ 0.05 (Table 4). Particularly for the Paraná coast feeding area, F_ST_ comparisons presented non-significant values for BA, ES, UB, AI, CB, and PB, whereas Φ_ST_ presented non-significant values for FN, RA, BA, ES, UB, AI, CB, and PB (Table 4).

**Table 3 ‒:** AMOVA values according to the feeding areas described for green sea turtles in the Atlantic Ocean, considering differences between the groups (North and South Atlantic), between feeding areas within the groups and between feeding areas overall.

AMOVA	
Between groups	64.53
Between feeding areas within the groups	7.97
Between individuals within the feeding areas	27.47

**Table 4 ‒:** F_ST_ according to haplotype frequency and Φ_ST_, using the Kimura 2P genetic distance parameters between the feeding areas described for green sea turtles in the Atlantic Ocean. F_ST_ is in the lower part of the table and Φ_ST_ in the upper part. Values in boldface and marked with an asterisk (*) indicate significant values (*p* ≤ 0.05). NC = North Carolina; FL = Florida; BH = Bahamas; NI = Nicaragua; BB = Barbados; CV = Cape Verde; AL = Almofala; FN = Fernando de Noronha; RA = Rocas Atoll; BA = Bahia; ES = Espírito Santo; UB = Ubatuba; PR = Paraná; AI = Arvoredo Island; CB = Cassino Beach; PB = Province of Buenos Aires.

	NC	FL	BH	NI	BB	CV	AL	FN	RA	BA	ES	UB	PR	AI	CB	PB
NC	-	0.04832	−0.00590	0.03620	0.23020*	0.73514*	0.57064*	0.73953*	0.64395*	0.76481*	0.79865*	0.77715*	0.83629*	0.78544*	0.78754*	0.77191*
FL	0.06507*	-	0.02679	−0.00617	0.35212*	0.81922*	0.66072*	0.81375*	0.73165*	0.86415*	0.87251*	0.86993*	0.90090*	0.87438*	0.88456*	0.86395*
BH	0.16787*	0.03877	-	0.00809	0.20367*	0.67557*	0.52953*	0.70266*	0.59373*	0.70080*	0.76703*	0.73244*	0.81499*	0.74232*	0.73876*	0.72378*
NI	0.24401*	0.09810*	0.01343	-	0.30436*	0.76501*	0.61871*	0.77808*	0.68374*	0.80259*	0.83977*	0.82430*	0.87639*	0.83088*	0.83545*	0.81623*
BB	0.05503*	0.13103*	0.18186*	0.27902*	-	0.32753*	0.14645*	0.37235*	0.21585*	0.33626*	0.45238*	0.36539*	0.51753*	0.38581*	0.36448*	0.36292*
CV	0.27769*	0.43270*	0.48096*	0.59643*	0.12231*	-	0.09570*	−0.01396	0.05324*	−0.03035	0.00683	0.10847*	0.07009*	0.01205	−0.02966	0.03239
AL	0.19327*	0.30512*	0.34087*	0.41797*	0.05419*	0.05428*	-	0.09228*	0.00343	0.08005*	0.11288*	0.05175*	0.10606*	0.05942*	0.03622	0.06051*
FN	0.26233*	0.39279*	0.42752*	0.50732*	0.11051*	−0.00940	0.03520	-	0.04527	−0.01836	0.00637	0.07376*	0.04556	0.00570	−0.02287	0.02126
RA	0.21361*	0.34459*	0.39034*	0.47796*	0.07083*	0.04290	−0.00464	0.02490	-	0.01993	0.04248	−0.00866	0.01138	−0.01232	−0.04492	−0.00304
BA	0.27135*	0.43090*	0.48707*	0.59264*	0.12351*	0.03327	0.01187	0.01563	−0.00308	-	−0.01482	0.09478*	0.03998	0.01252	0.00529	0.01732
ES	0.29830*	0.43096*	0.46541*	0.53585*	0.14773*	0.05119*	0.01908	0.03136	0.00717	−0.01064	-	0.05569*	0.01473	−0.00353	−0.02433	0.00538
UB	0.37569*	0.52883*	0.57126*	0.64876*	0.24937*	0.21737*	0.08079	0.16262*	0.07996*	0.07014*	0.05047*	-	0.01306	0.02195	0.01856	0.01225
PR	0.37084*	0.49934*	0.52877*	0.58776*	0.23065*	0.16037*	0.06056*	0.12100*	0.05546*	0.03577	0.02429	0.00199	-	−0.00061	−0.00974	−0.00406
AI	0.30735*	0.44978*	0.49191*	0.56738*	0.16606*	0.10014*	0.02736	0.06917*	0.01869	0.00168	0.00152	0.01657	0.00203	-	−0.01648	−0.00978
CB	0.30733*	0.45348*	0.49871*	0.57670*	0.16984*	0.10955*	0.03076	0.07657*	0.02421	0.00696	0.00514	0.01275	0.00060	−0.00770	-	−0.02359
PB	0.32169*	0.47217*	0.51756*	0.59893*	0.18197*	0.11329*	0.03551	0.08002*	0.02583	0.00778	0.00471	0.01146	−0.00170	−0.00749	−0.00759	-

## Discussion

A total of 12 haplotypes were identified among the 285 juvenile green turtles sampled along the Paraná coast, Southern Brazil. A predominance of the haplotype CMA8, concerning both number of individuals and continuous presence over the years, reinforces the genetic characteristics recorded in different regions over the Southwestern Atlantic Ocean. The number of haplotypes and the genetic diversity values found for the individuals sampled between 2007 and 2014 highlight the relevance of this region as a feeding and development ground for multiple genetic stocks of juvenile green turtles from the South Atlantic Ocean.

Considering the individuals from the Paraná coast by sampling year, the second most frequent haplotype (CM-A5) was observed for all years except 2007, and the other 10 haplotypes occurred with a relatively low frequency (≤ 5%). Three haplotypes occurred more frequently on the Paraná coast than in the other previously described Atlantic feeding areas; haplotype CMA42 was identified in six individuals, whereas haplotypes CMA39 and CMA46 were identified only in Brazil, once in Arvoredo Island (Santa Catarina state) and once in Ubatuba (São Paulo state), respectively (Naro-Maciel et al., 2007; Proietti et al., 2012). These differences in haplotype occurrence may be associated with biogeographic and migratory behavior, affected by climate and oceanographic conditions. The Paraná coast is humid mesothermal (subtropical) and is influenced by polar air masses in winter and by tropical Atlantic masses in summer (Vanhoni and Mendonça, 2008). These variations, including the food availability and coastal water mass and currents, might influence the presence of animals from different reproductive stocks in feeding areas (Proietti *et al*., 2009; Prosdocimi et al., 2012). 

The haplotypes analyzed in this study are identified mainly from nesting areas in the South Atlantic (coast of Brazil and Africa) (Formia et al., 2006; Naro-Maciel et al., 2007; Proietti et al., 2009; Proietti *et al*., 2012) and only three of them (CM-A3, CM-A5 and CM-A6) are described for nesting areas in the North Atlantic and the Caribbean (Encalada et al., 1996; Naro-Maciel *et al*., 2007; Proietti *et al*., 2009). Future studies addressing biogeographic issues should correlate genetic diversity and oceanographic and climatic factors. This approach would make an important contribution to understanding the potential effects of climate change on mixed stocks using specific foraging grounds.

Overall, 10 haplotypes were identified with low frequency in the present study; this finding is consistent with other studies conducted along the Brazilian coast (Naro-Maciel et al., 2007; Naro-Maciel *et al*., 2012; Proietti et al., 2012; Jordão et al., 2015). Considering *h* and π values, this study presents results similar to those presented for other feeding areas across the whole Atlantic Ocean (Bass et al., 1998; Lahanas et al., 1998; Bass and Witzell, 2000; Luke et al., 2004; Bass *et al*., 2006; Naro-Maciel *et al*., 2007, 2012; Monzón-Argüello et al., 2010; Proietti *et al*., 2012; Prosdoscimi *et al*., 2012). Comparing the results of the present study with those of a previous study conducted on the Paraná coast between 2005 and 2008 (Jordão *et al*., 2015), the diversity of haplotypes increased: six haplotypes were identified in both studies, six are new for this region, and only one was not sampled again (CM-A1). These results reinforce the importance of this feeding area for multiple stocks of green turtles, and also that maintaining management and conservation actions is crucial to guarantee the genetic variability along the Southwestern Atlantic Ocean.

The difference between the North and South Atlantic groups (AMOVA between groups of 64.56%) may be associated with the geographical distances between the feeding areas and the influence of sea currents; the relatively warm currents that flow off the Brazilian coast (Equatorial Atlantic and Central South Atlantic) form a direct front against the cold-water Malvinas Current (Tomczak and Godfrey, 2003). These different currents interfere with the presence of individuals in the feeding areas because, reportedly, individuals remain on the coast of Argentina and Uruguay during summer and autumn and migrate to Southern Brazil where waters are warmer in winter (Carman et al., 2012). The values found for genetic differences could also result from colonization events that influence the distribution of animals among nesting areas and the location of the feeding areas (Bass et al., 2006).

In studies on South Atlantic feeding areas, non-significant values were observed when comparing the data with other areas geographically close to the Paraná coast, which was expected because these regions have similar conditions in terms of the influence of sea currents and other environmental factors. Similar results were observed for feeding areas of the Province of Buenos Aires on the coast of Argentina, which presented significant differences when compared with the five feeding areas in the Northeastern Atlantic, but also with one northern area in the Southwestern Atlantic Ocean (Prosdoscimi *et al*., 2012). Moreover, on the Brazilian coast, two feeding areas in Southern Brazil (Rio Grande do Sul state) exhibited genetic differences when compared with the North Atlantic areas, but no differences from Ubatuba and Rocas Atoll in Southeastern and Northeastern Brazil (Proietti et al., 2012). The composition of feeding areas might depend on the movement of individuals between feeding areas, the abundance of nesting sites, the distances between the nesting and feeding areas, philopatry, and sea currents (Prosdoscimi *et al*., 2012). 

According to Wright’s scale (1978), F_ST_ and Φ_ST_ values are significant when considering the comparisons between the Paraná coast and North Atlantic areas, and the comparisons with genetic difference values are either high or very high. Comparing the Paraná coast with other South Atlantic areas, F_ST_ had significant values for AL, FN, and RA, and Φ_ST_ had significant values for AL, but genetic difference values were either moderate or small. The genetic difference values obtained between the Paraná coast and other regions could be the result of oceanographic parameters, considering that the sea currents may favor individuals following the direction of the south coast of Brazil or move them away from Brazil with other predominant currents (Naro-Maciel et al., 2007). Phylogeographic analysis in nesting grounds of green turtles distinguished two haplotype groups (Northwestern and Southwestern), and the pattern of distribution is non-random among feeding areas and could be influenced by ocean currents (Prosdocimi et al., 2012).

Concerning the samples in the present study according to the sampling year, on average, five haplotypes were identified per year, but the occurrence of haplotype CMA23 was novel. This haplotype is exclusively described for South Atlantic nesting areas (Ascension Island and Rocas Atoll/Fernando de Noronha) (Proietti et al., 2012). The occurrence of this haplotype brings new information on this feeding area and for the entire Regional Management Unit (RMU). By updating information about the species in the RMU, it is possible to improve the protection of the nesting populations, since this considers the overlapping of areas of nesting and feeding, regarding reproduction and threats (Wallace et al., 2011). The identification of the occurrence of *Chelonia mydas* haplotypes in the foraging areas enables identification of the presence of mixed stocks, which makes the foraging areas important in terms of conservation, since their preservation ensures that a suitable place is maintained for the occurrence of individuals from different nesting areas. This survey can be considered in the elaboration of plans for monitoring water quality, food availability, vessel traffic, installations of new projects, and other modifications that may change the conditions of the Paraná coast in the future.

Collectively, the results of the included samples from 2007 to 2014 along the Paraná coast, support the use of this specific foraging ground by juvenile *Chelonia mydas* from multiple origins, and evidence its considerable genetic variability. The study also highlights the presence of different haplotypes in different years and the importance of developing systematic and continuous monitoring of feeding areas to understand connectivity and migratory patterns. This variation over the years may be related to the sea currents, climatic issues, or other oceanographic characteristics; these and other factors should also be investigated more carefully in the future. Furthermore, future studies should include analysis using different molecular markers to improve knowledge on turtle origins and biogeography. In general, the results contribute crucial information to improve management and conservation plans for *Chelonia mydas* and its habitats.

## References

[B1] Abreu-Grobois A, Horrocks J, Formia A, Dutton P, Leroux R, Vélez-Zuazo X, Soares L, Meylan P (2006). New mtDNA Dloop primers which work for a variety of marine turtle
species may increase their solution of mixed stock analyses.

[B2] Almeida AP, Santos AJB, Thomé JCA, Belini C, Baptistotte C, Marcovaldi MÂ, dos Santos ASS, Lopez M (2011). Avaliação do estado de conservação da tartaruga marinha Chelonia
mydas (Linnaeus, 1758) no Brasil. BioBrasil.

[B3] Almeida FS, Fungaro MHP, Sodré LMK (2001). RADP and isoenzyme analysis of genetic variability in three
allied species of catfish (Siluriformes: Pimelodidae) from the Tibagi
river. J Zool.

[B4] Andrade MF, Domit C, Broadhurst MK, Tolhurst DJ, Silva-Souza ÂT (2016). Appropriate morphometrics for the first assessment of juvenile
green turtle (Chelonia mydas) age and growth in the south-western
Atlantic. Mar Biol.

[B5] Bass AL, Lagueux CJ, Bowen BW (1998). Origin of green turtles, Chelonia mydas, at “Sleeping Rocks” off
the northeast coast of Nicaragua. Copeia.

[B6] Bass AL, Witzell WN (2000). Demographic composition of immature green turtles (Chelonia
mydas) from the east central Florida coast: Evidence from mtDNA
markers. Herpetologica.

[B7] Bass AL, Epperly SP, Braun-Mcneill J (2006). Green turtle (Chelonia mydas) foraging and nesting aggregations
in the Caribbean and Atlantic: Impact of currents and behavior on
dispersal. J Hered.

[B8] Bowen BW, Karl SA (2007). Population genetics and phylogeography of sea
turtles. Mol Ecol.

[B9] Bresette MJ, Witherington BE, Herren RM, Bagley DA, Gorham JC, Traxler SL, Crady CK, Hardy R (2010). Size-class partitioning and herding in a foraging group of green
turtles Chelonia mydas. Endanger Species Res.

[B10] Cantor M, Barreto AS, Taufer RM, Giffoni B, Castilho PV, Maranho A, Beatriz C, Kolesnikovas C, Godoy D, Rogério DW (2020). High incidence of sea turtle stranding in the southwestern
Atlantic Ocean. ICES J Mar Sci.

[B11] Carman VG, Falabella V, Maxwell S, Albareda D, Campagna C, Mianzan H (2012). Revisiting the ontogenetic shift paradigm: The case of juvenile
green turtles in the SW Atlantic. J Exp Mar Biol Ecol.

[B12] Coelho VF, Domit C, Broadhurst MK, Prosdocimi L, Nishizawa H, Almeida FS (2018). Intra-specific variation in skull morphology of juvenile Chelonia
mydas in the southwestern Atlantic Ocean. Mar Biol.

[B13] Encalada SE, Lahanas PN, Bjorndal KA, Bolten AB, Miyamoto MM, Bowen BW (1996). Phylogeography and population structure of the Atlantic and
Mediterranean green turtle Chelonia mydas: A mitochondrial DNA control
region sequence assessment. Mol Ecol.

[B14] Excoffier L, Lischer HEL (2010). Arlequin suite ver 3.5: A new series of programs to perform
population genetics analyses under Linux and Windows. Mol Ecol Resour.

[B15] Formia A, Godley BJ, Dontaine J-F, Bruford MW (2006). Mitochondrial DNA diversity and phylogeography of endangered
green turtle (Chelonia mydas) populations in Africa. Conser Genet.

[B16] Fuentes MMPB, Wildermann N, Gandra TBR, Domit C (2020). Cumulative threats to juvenile green turtles in the coastal
waters of southern and southeastern Brazil. Biodivers Conserv.

[B17] Gama LR, Domit C, Broadhurst MK, Fuentes MMPB, Millar RB (2016). Green turtle Chelonia mydas foraging ecology at 25º S in the
western Atlantic: Evidence to support a feeding model driven by intrinsic
and extrinsic variability. Mar Ecol Prog Ser.

[B18] Geraci JR, Lounsbury VJ (2005). Marine mammals ashore: A field guide for strandings.

[B19] Guebert-Bartholo FM, Barletta M, Costa MF, Monteiro-Filho ELA (2011). Using gut contents to assess foraging patterns of juvenile green
turtles Chelonia mydas in the Paranaguá Estuary, Brazil. Endanger Species Res.

[B20] IUCN (2019). Chelonia mydas (South Atlantic subpopulation). The IUCN Red List of Threatened Species 2019.

[B21] Jensen MP, Limpus CJ, Whiting SD, Guinea ML, Prince RIT, Dethmers KEM, Adnyana W, Kennett R, FitzSimmons NN (2013). Defining olive ridley turtle Lepidochelys olivacea management
units in Australia and assessing the potential impact of mortality in ghost
nets. Endanger Species Res.

[B22] Jordão JC, Bondioli ACV, Guebert FM, Thoisy BD, Toledo LFA (2015). Green turtle (Chelonia mydas) genetic diversity at Paranaguá
estuarine complex feeding grounds in Brazil. Genet Mol Biol.

[B23] Kimura M (1980). A simple method for estimating evolutionary rates of base
substitutions through comparative studies of nucleotide
sequences. J Mol Evol.

[B24] Lahanas PN, Bjorndal KA, Bolten AB, Encalada SE, Miyamoto MM, Valverde RA, Bowen BW (1998). Genetic composition of a green turtle (Chelonia mydas) feeding
ground population: Evidence for multiple origins. Mar Biol.

[B25] Lana PC, Marone E, Lopes RM, Machado EC, Seeliger U, Kjerfve B (2000). The subtropical Estuarine Complex of Paranaguá Bay,
Brazil. Coastal marine ecosystems of Latin America.

[B26] Lenz AJ, Avens L, Borges-Martins M (2017). Age and growth of juvenile green turtles Chelonia mydas in the
western South Atlantic Ocean. Mar Ecol Prog Ser.

[B27] Librado P, Rozas J (2009). DnaSP v5: A software for comprehensive analysis of DNA
polymorphism data. Bioinformatics.

[B28] López-Barrera EA, Longo GO, Monteiro-Filho ELA (2012). Incidental capture of Green turtle (Chelonia mydas) in gillnets
of small-scale fisheries in the Paranaguá Bay, Southern
Brazil. Ocean Coast Manage.

[B29] Luke K, Horrocks JA, Leroux RA, Dutton PH (2004). Origins of green turtle (Chelonia mydas) feeding aggregations
around Barbados, West Indies. Mar Biol.

[B30] Mazaris AD, Matsinos G, Pantis JD (2009). Evaluating the impacts of coastal squeeze on sea turtle
nesting. Ocean Coast Manage.

[B31] Miranda TP, Genzano GN, Marques AC (2015). Areas of endemism in the Southwestern Atlantic Ocean based on the
distribution of benthic hydroids (Cnidaria: Hydrozoa). Zootaxa.

[B32] Monteiro DS, Estima SC, Gandra TBR, Silva AP, Bugoni L, Swimmer Y, Seminoff JA, Secchi ER (2016). Long-term spatial and temporal patterns of sea turtle strandings
in southern Brazil. Mar Biol.

[B33] Monzón‐Argüello C, López‐Jurado LF, Rico C, Marco A, López P, Hays GC, Lee PLM (2010). Evidence from genetic and Lagrangian drifter data for
transatlantic transport of small juvenile green turtles. J Biogeogr.

[B34] Musick JA, Limpus CJ, Lutz PL, Musick JA (1997). Habitat utilization and migration in juvenile sea
turtles. The biology of sea turtle.

[B35] Naro-Maciel E, Becker JH, Lima EHSM, Marcovaldi MA, DeSalle R (2007). Testing dispersal hypotheses in foraging green sea turtles
(Chelonia mydas) of Brazil. J Hered.

[B36] Naro-Maciel E, FitzSimmons MLNN, Amato G (2008). Evolutionary relationships of marine turtles: A molecular
phylogeny based on nuclear and mitochondrial genes. Mol Phylogenet Evol.

[B37] Naro-Maciel E, Bondioli ACV, Martin M, De Pádua Almeida A, Baptistotte C, Bellini C, Marcovaldi MA, Santos AJB, Amato G (2012). The interplay of homing and dispersal in green turtles: A focus
on the southwestern Atlantic. J Hered.

[B666] Naro-Maciel E, Reid BN, Alder SE, Amato G, Bjorndal KA, Bolten AB, Martin M, Nairn CJ, Shamblin B, Pineda-Catalan O (2014). From refugia to rookeries: phylogeography of Atlantic green
turtles. J Exp Mar Biol Ecol.

[B38] Naro‐Maciel E, Hart KM, Cruciata R, Putman NF (2016). DNA and dispersal models highlight constrained connectivity in a
migratory marine megavertebrate. Ecography.

[B39] Proietti MC, Lara-Ruiz P, Reisser JW, Pinto LDS, Dellagostin AO, Marins LF (2009). Green turtles (Chelonia mydas) foraging at Arvoredo Island in
Southern Brazil: Genetic characterization and mixed stock analysis through
mtDNA control region haplotypes. Genet Mol Biol.

[B40] Proietti MC, Reisser JW, Kinas PG, Kerr RD, Monteiro DS, Marins LFF, Secchi ER (2012). Green turtle Chelonia mydas mixed stocks in the western South
Atlantic, as revealed by mtDNA haplotypes and drifter
trajectories. Mar Ecol Prog Ser.

[B41] Prosdocimi L, Carman VG, Albareda DA, Remis MI (2012). Genetic composition of green turtle feeding grounds in coastal
waters of Argentina based on mitochondrial DNA. J Exp Mar Biol Ecol.

[B42] Sanches TM (1999). Avaliação e ações prioritárias para a conservação da biodiversidade da
zona costeira e marinha: tartarugas marinhas. Termo de Referência
nº155/98.

[B43] Seminoff JA (2004). Chelonia mydas. The IUCN Red List of Threatened Species.

[B44] Shamblin BM, Bjorndal KA, Bolten AB, Hillis‐Starr ZM, Lundgren IAN, Naro-Maciel E, Nairn CJ (2012). Mitogenomic sequences better resolve stock structure of southern
Greater Caribbean green turtle rookeries. Mol Ecol.

[B45] Tagliolatto AB, Goldberg DW, Godfrey MH, Monteiro‐Neto C (2019). Spatio‐temporal distribution of sea turtle strandings and factors
contributing to their mortality in south‐eastern Brazil. Aquat Conserv.

[B46] Tamura K, Stecher G, Peterson D, Filipski A, Kumar S (2013). MEGA6: Molecular Evolutionary Genetics Analysis version
6.0. Mol Biol Evol.

[B47] Togawa RC, Brigido MM (2003). PHPH: Web based tool for simple electropherogram quality
analysis.

[B48] Tomczak M, Godfrey JS (2003). Regional oceanography: An introduction.

[B49] Vanhoni F, Mendonça F (2008). O clima do litoral do estado do Paraná. Rev Bras Climatol.

[B50] Wallace BP, Dimatteo AD, Bolten AB, Chaloupka MY, Hutchinson BJ, Abreu-Grobois FA, Mortimer JA, Seminoff JA, Amorocho D, Bjorndal KA (2011). Global conservation priorities for marine turtles. PLoS One.

[B51] Wildermann NE, Gredzens C, Avens L, Barrios-Garrido HA, Bell I, Blumenthal J, Bolten AB, McNeill JB, Casale P, Domenico MD (2018). Informing research priorities for immature sea turtles through
expert elicitation. Endanger Species Res.

[B52] Wright S (1978). Evolution and the genetics of populations volume 4: Variability within
and among natural populations.

[B53] Wyneken J (2001). The anatomy of sea turtles. U.S. Department of Commerce NOAA Technical
Memorandum NMFS-SEFSC-470, Miami, Florida.

